# Supplementary material for: Immobility of isolated swarmer cells due to local liquid depletion
Source: ArXiv. 2024 Nov 26:arXiv:2411.17842v1. Preprint. [Version 1] (PMC11623706)
Supplement: Supplement 1 [file NIHPP2411.17842v1-supplement-1.pdf]

Supplementary Information

Movie S1. A real-time, phase-contrast movie of swarming cells near the colony edge, with tiny MgO smoke beads gently deposited on the colony. Beads outside the colony are immobile. Cell-size is ~1×7 μm.

Movie S2. A phase-contrast movie of swarming cells inside the colony (100 μm away from the edge), with tiny MgO smoke beads gently deposited on the colony. Beads move fast close to temporarily immobile cells, indicating that there is flowing liquid near the stationary cells. Cell size is ~1×7 μm; frame rate is reduced by a factor of 25.

Movie S3. A real-time movie showing fluorescence labelling of flagella in swarming cells. Only the flagella are labelled, the cell-bodies are invisible. The fraction of labelled cells is 1/20 (there are many more cells on the agar but they are not shown).

Appendix 1 – TL DIC microscopy

In the current work we use Transmitted-Light Differential Interference Contrast (TL DIC) microscopy equipped with color "enhancement". As in all color-DIC systems, the incident light travels through a polarizer, a Rochon/Wollaston/Nomarski prism that splits the ray, and a lambda plate. In our case, the light hits the sample from below, the bottom side of the sample is flat and smooth, and the substrate does not have birefringent properties (the bottom of the Petri-dish is made of a special glass and not the standard plastic).

Loosely speaking, the color-DIC system can differentiate between slopes in the sample, which are manifested in the hue property of colors. Differences in the index of refraction between the cells and the water (liquid), which are typically smaller than 0.05, do not play a role in determining the obtained hue, specifically in our systems where the cells are thin; Within the tested range, the same hue will be obtained for all objects in the sample if they have the same slope. In non-color DIC for instance, the differences in the index of refraction are resulted in an almost complete destructive interference, and only the perimeter of the cells may be resolved. This means that in color-DIC, cells embedded in water are almost invisible (in contrast e.g., to phase-contrast microscopy where cells embedded in water are nicely resolved).

Overall, within the experimental conditions and range of the slopes measured, the obtained hue is a monotonic function of only the local slopes in the upper surface and not of the material through which the light is transmitted. Mind that the saturation and luminance do depend on the material (its thickness or density for instance) through which the light is transmitted so the cells may sometimes be seen e.g., in Fig. 4c.

The lateral resolution (in the viewing plane) of DIC is of the order of the wavelength i.e., ~0.5 μm (stemming from the 0.2 μm adjutant points of the two split rays), and the ability to resolve the vertical axis by the hue is typically of the order of 0.05 μm, which yields an approximate minimal "local" angle of detection of ~4° (i.e., slopes smaller than 4° are not easily resolved; there is also a maximal angle above which the light is reflected away from the lens and the sample becomes dark).

In order to relate between the liquid around a cell and the measured hue value, consider a cross section of a bacterium with a cylindrical shape with a diameter 1 μm, resting on a flat surface (see Fig. 7a). Liquid (e.g., water) may cover the cell completely or partially. In Fig. 7b, the cell is completely covered. The height of the water is not important – only the angle it creates with

respect to the tangent to the “ridge” of the cell. Thus, all samples that are covered by a flat-water interface will yield the same hue, regardless of the water height. In Fig. 7c we show a different case where the liquid does not completely cover the cell, resulting in different interfacial slopes around the cell. The ridge of the cell is parallel to the background and will yield the same hue. However, the edges of the cell are covered with liquid that creates a changing slope; the local slope will yield a hue that reflects the slope's value. In Figs. 7d-e, the layer of liquid is shallow, or there is no liquid at all. In these cases, only the ridge of the cell has a parallel surface that will yield the same hue as the one seen for the background. The edges of the cell will have different hues depending on their local slopes, independently of the liquid. Upon increasing the liquid layer, the system transitions from Figs. 7d-e to Fig. 7c or even Fig. 7b, and the slopes of the liquid on the cell edges become moderate, which changes the hue to the background value. Note that the ridge of the cell is very narrow; it is narrower than the lateral resolution – therefore, the background hue is seldom obtained on the ridge.

To illustrate how DIC works on transparent objects embedded in transparent medium, we performed an experiment described next and documented in Fig. 8. In Fig. 8a we show a set of images of spherical silica beads (5 μm in diameter) deposited on an agar surface. A drop of water, mixed with surfactant, is allowed to spread over the agar. The surfactant is added to form a relatively thin liquid front. The phase-contrast images are presented in order to show the advancing edge of the drop that covers the beads. The images show that the beads are nicely resolved with the water on top of them, and that they are not being flashed by the liquid (they are slightly embedded in the agar and a slight motion might be detected as the agar is rewetted). The focus is adjusted after the water covers the beads. The DIC set of images (Fig. 8b), shows a silica bead and the different colors (hues) that are exhibited along a diagonal line parallel to the beam splitting direction (from top-left to bottom-right). The hues reflect the structure of both the bead and the surface on which it is deposited. The top of the bead has the same hue as the one seen at the background (pale-blue). The intensity of the outer regions of the bead are low due to the relatively large angles, as most light is reflected away from the objective lens. The reddish and yellowish colors along the beam splitting direction, but outside of the bead, indicate that the bead is slightly embedded in the agar. When water approaches, the bead is completely covered with water, and the surface becomes flat and smooth and almost parallel to the agar (except for the advancing contact angle which is very small ~ 7°; it thus appears brown and not pale-blue). In contrast to the phase-contrast case, in the DIC images the bead is completely invisible – even though we travel with the stage to find the appropriate focus. After

a few minutes (the process is gradual but may be fairly seen after few minutes), the surface becomes pale-blue again because the angle of the drop decreases. The experiment lasts about 20 min during which the water evaporates and absorbed by the agar (and the bead drifts a bit with respect to the lab because the soft agar is not stable enough during the “flood”). During capturing, we play with the focus every few seconds to show that the bead cannot be detected. The bead sometimes appears as a pale ring with a dot at its center, but only when the water level is low enough, the colors reappear.

Appendix 2 - flagella staining

Our protocol is similar to other protocols recently used to stain flagellar filaments in *Bacillus subtilis* 3610 and other species[63,64]; yet, in our case the cells are swarmers. In the strain we have used (DS1916, amyE::Phag-hagT209C spec), the flagellin protein genes have been modified to include the cysteine amino acid that binds to the maleimide functional group, allowing staining using Alexa 546 dye (ThermoFischer ref A10258). Bacteria are grown overnight (~18 h) in 2 ml of LB (25 g/l), at 30°C and shaking (200 rpm), from which 10 µl is used to inoculate three swarm plates (LB with 0.5% agar). When the droplets dry out, the plates are placed in an incubator at 30°C with controlled high humidity (~95% RH). Additionally, a fourth plate is inoculated with a one-hour delay. This plate will be used to reincorporate the stained bacteria into swarming conditions. To facilitate that process, the inoculation is done slightly off-center. The remaining overnight culture is centrifuged for 3 minutes at 2000g, and the supernatant is saved for later.

The three initial plates are incubated for five hours, after which the bacteria are collected by flushing the colonial edge with phosphate buffer (0.01 M ph~7.2) and then collecting the liquid with the cells. Special attention must be put into collecting bacteria exclusively from the edge, where bacteria are in the swarming state. If not, bio-aggregates are created by bacteria in the biofilm, disturbing the following steps. After collection, flagella are stained by adding 5 µl of the dye solution (20 µg/µl of Alexa 546 in DMSO). We allow staining to occur for one hour while the suspension is rotated at 100 rpm to prevent bacterial sedimentation. Then, bacteria are centrifuged for 3 minutes at 2000g and resuspended in the overnight supernatant (0.2 µm filtered). Then, 20 µl is inoculated into the fourth agar plate. The inoculation drop is introduced outside of the colony, and then it spreads along the boundary of the colony, forming an arc, never being in direct contact with the colony. After drying, the plate is replaced in the incubator for 30 minutes or until the colony integrates with the stained bacteria. At the end of this process, one will obtain a swarming colony where a fraction of the cells has stained flagella.

Observation was done using the same fluorescence setup described above (Zeiss Axio Imager Z2 at 63×, filter set 20 Rhodamin shift free: Excitation 546/12; Beam Splitter 560; Emission 607/80), and a Zyla Andor operated at 100 frames per second.

Appendix 3 – image analysis

The quantitative analysis presented in Figs. 2 and 3 is facilitated using custom image analysis software implemented in Matlab. First, images undergo standard smoothing and preprocessing, as detailed in[41]. Applying a threshold on the intensity yields a Boolean mask indicating whether each pixel corresponds to a cell or not. Occupancy statistics (Fig. 2) corresponds to pixel-wise counts (and not number of cells).

The sequence of images is used to obtain velocity field using optical flow analysis (see[31] for details). Such algorithms inherently involve smoothing. Average speeds (Figs. 3ab) are obtained directly from the vector fields without taking into account speeds below a threshold. In other words, we average only over pixels that correspond to moving objects. The implementation follows[41].

Figures. 3cd show the average speed of cells as a function of the number of neighbors (up to a given distance). This requires identifying the position of individual cells and therefore tracking of individuals. To this end, we apply a novel method for tracking elongated objects using the Hough transform. First, the Boolean mask is separated into connected blobs using standard image analysis tools in Matlab. Blobs that are too small (in area or length) are discarded. The main difficulty is that cells that are practically touching are often associated with the same blob. To bypass this problem, in each blob, we find the longest line segment using the Hough transform. This segment (with some added thickness) is removed from the blob. Segments that are longer than 1.5 the average cell length are split in two. Repeating the process, we obtain a list of line segments that correspond to the individual cells.

Once individual cells were identified as line segments, we need to associate cells in consecutive frames to obtain trajectories. Typically, tracking software use the central points to find which cell in one image is closest to which cells in the following one. Here, we use the sum of the distances between the endpoints, which also takes into account the orientation and length of the detected cells. Since the order of the endpoints in each line segment is arbitrary, one needs to take the minimum between two ordering options. We perform a standard greedy pair matching algorithm, starting at the closed pair up to a cutoff distance of about 3 average cell lengths.

Next, the endpoints of each trajectory are smoothed over time using malowess with a linear interpolation. Velocities of the center of mass can then be calculated. Finally, to calculate the number of neighbors a focal cell has up to a given distance, we find the average number of

endpoints associated with other cells to each of the endpoints of the focal cell. In other words, instead of counting cells, we count endpoints, each "worth" half a cell. This counting is used to calculate the statistics in Figs. 3cd.
